# Infection–induced Bystander-Apoptosis of Monocytes Is TNF-alpha-mediated

**DOI:** 10.1371/journal.pone.0053589

**Published:** 2013-01-17

**Authors:** Stephan Dreschers, Christian Gille, Martin Haas, Julia Grosse-Ophoff, Marion Schneider, Anja Leiber, Hans-Jörg Bühring, Thorsten W. Orlikowsky

**Affiliations:** 1 Department of Neonatology, University Children’s Hospital, Aachen, Germany; 2 Department of Neonatology, University Children’s Hospital, Tuebingen, Germany; 3 Department of Experimental Anesthesiology, University Hospital, Ulm, Germany; 4 FACS Core Facility, University Hospital, Tuebingen, Germany; University of Tübingen, Germany

## Abstract

Phagocytosis induced cell death (PICD) is crucial for controlling phagocyte effector cells, such as monocytes, at sites of infection, and essentially contributes to termination of inflammation. Here we tested the hypothesis, that during PICD bystander apoptosis of non-phagocyting monocytes occurs, that apoptosis induction is mediated via tumor necrosis factor-alpha (TNF-α and that TNF-α secretion and -signalling is causal. Monocytes were infected with *Escherichia coli* (*E. coli*), expressing green fluorescent protein (GFP), or a pH-sensitive Eos-fluorescent protein (EOS-FP). Monocyte phenotype, phagocytic activity, apoptosis, TNF-receptor (TNFR)-1, -2-expression and TNF-α production were analyzed. Apoptosis occured in phagocyting and non-phagocyting, bystander monocytes. Bacterial transport to the phagolysosome was no prerequisite for apoptosis induction, and desensitized monocytes from PICD, as confirmed by EOS-FP expressing *E. coli. C*o-cultivation with non-infected carboxyfluorescein-succinimidyl-ester- (CFSE-) labelled monocytes resulted in significant apoptotic cell death of non-infected bystander monocytes. This process required protein *de-novo* synthesis and still occurred in a diminished way in the absence of cell-cell contact. *E. coli* induced a robust TNF-α production, leading to TNF-mediated apoptosis in monocytes. Neutralization with an anti-TNF-α antibody reduced monocyte bystander apoptosis significantly. In contrast to TNFR2, the pro-apoptotic TNFR1 was down-regulated on the monocyte surface, internalized 30 min. p.i. and led to apoptosis predominantly in monocytes without phagocyting bacteria by themselves. Our results suggest, that apoptosis of bystander monocytes occurs after infection with *E. coli* via internalization of TNFR1, and indicate a relevant role for TNF-α. Modifying monocyte apoptosis in sepsis may be a future therapeutic option.

## Introduction

Monocytes and granulocytes are part of the host’s rapid response component generating a vigorous antibacterial reaction upon contact with microbes. The first steps of effectively eliminating bacteria are binding and phagocytosis. These processes are accompanied by cellular and humoral host cell signals. A key role in the orchestration of an antibacterial host response plays a phenomenon, called phagocytosis-induced cell death (PICD), provoking effector cell apoptosis related to phagocyosis and through this contributing to a controlled termination of inflammation [1]. Thus, bacterial phagocytosis plays a dual role: Elimination of bacteria and termination of inflammation. It is conceivable that a dysregulation or imbalance of PICD in the host is accompanied with problems: If, on the one side, monocytes undergo abortive PICD, bacteria may be eliminated incompletely. On the other side, in case of delayed or insufficient PICD, permanent or prolonged cytokine production via activated effector cells could be another extreme, in which inflammation becomes systemic (Systemic Inflammation Response Syndrome, SIRS), and the host may be harmed. The clinical picture of sepsis is characterized by a hyperinflammatory state with SIRS followed by a state of immunoparalysis, called compensatory anti-inflammatory syndrome (CARS). Most experimental clinical therapies in adults [2], children [3], and neonates [4] have focused on attenuating the initial inflammatory response, possibly exacerbating the progressive development of immunosuppression [1]. Although these approaches have demonstrated modest benefits in select patient groups, the majority of deaths occur in patients with sepsis who are immune suppressed. This immunosuppressive phase is characterized by loss of delayed type hypersensitivity response to control antigens, failure to clear the primary infection and development of new secondary infections [1]. A crucial role for this step in the pathogenesis of sepsis is an early and ongoing apoptotic depletion of cells of both the innate and adaptive immune system [5]. Uptake of apoptotic cells further impairs host immunity by inducing an anti-inflammatory phenotype in phagocytic cells that consume the cellular corpses [6], [7], [8]. Prevention of this sepsis-induced apoptosis apparently attenuates the immunosuppressive cascade and leads to sustained immunity.

TNF-α, a potent inflammatory cytokine, plays an important role in immunity to numerous bacterial infections. It acts through members of the TNF receptor (TNFR) family, and its initial form is a 26-kDa transmembrane protein (mTNF). After cleavage from the cell surface by a metalloproteinase, TNF-α is subsequently released as a 17-kDa protein. After trimerization it binds to two receptors: TNFR1 and TNFR2. TNF-α stimulates inflammation by activating multiple gene transcription, but also supports both, pro- and anti-apoptotic signals (reviewed in [9], [10], [11]). Apoptosis is mediated via binding of TNF-α to the TNFR1 receptor. Once the ligand has bound the receptor is internalized and recruits adapter proteins required for pro-apoptotic signalling [12]. The pro-apoptotic pathway is activated upon endocytosis of the TNF-α TNF receptor complex [9]; [11], [13], [14], whereas binding of TNF-α to TNF receptors on the outer membrane skews to anti-apoptotic signalling [9]. Contact to a pathogen may be sufficient for monocytes to secrete TNF-α [15], [16], [17], [18].

The Fas/Fas-ligand system (CD95/C95L) was shown to be relevant for induction of PICD [19]. CD95 and CD95L belong to the TNF-family and TNF-α receptor/ligand interaction may trigger pro- and anti-apoptotic signals [9], [20], [21], [22]. Pharmacological inhibition of phagocytosis reduced the induction of PICD [23], [24], suggesting different steps of phagocytosis to be a prerequisite for PICD in monocytes. Therefore we analyzed different stages of bacterial phagocytosis by monocytes with regard to occurrence of cell death and the involvement of TNF-α.

## Results

### *E. coli* Induce Monocyte Apoptosis Early after Infection

In previous studies, we described PICD of monocytes after phagocytosis of *E. coli* or *Streptococci*, which had been purified from adults and from cord blood samples [23]. Here we infected monocytes in cultures of peripheral blood mononuclear cells (PBMC) with *E. coli*-GFP and detected apoptosis four hours *post infectionem* (p.i.). The phagocytosis index of monocytes, defined as CD14-positive cells, was 66.8±13.1% and comparable to previous results with purified monocytes.

**Figure 1:**
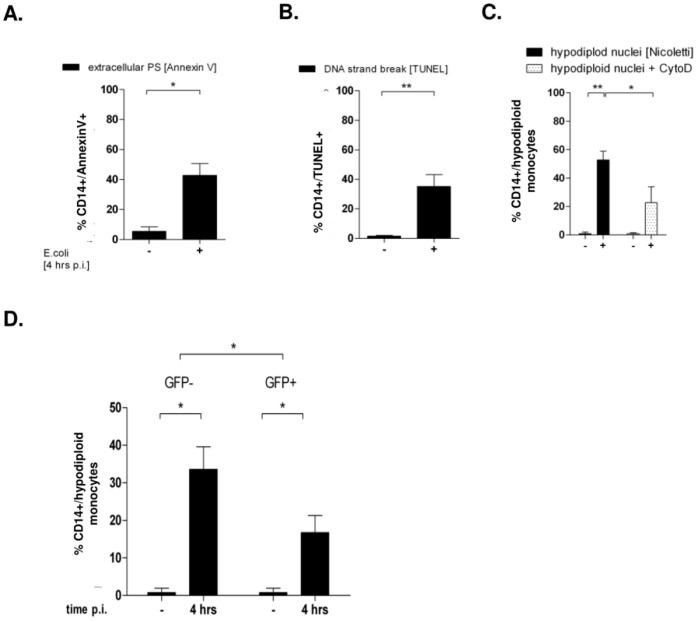
Apoptosis occurs after infection with *E. coli*-GFP 4 h p.i. in phagocyting and non-phagocyting monocytes. PBMC were infected with *E. coli*-GFP for 4 hours and free bacteria were removed. When indicated, CytoD was supplemented 30 minutes prior to infection. Monocyte apoptosis was detected by annexin V (A; n = 5), DNA strand breaks (B; n = 7), and hypodiploidity (Fig. 1C, n = 14, * p<0.05, ** p<0.01). In the same experimental setup, CD14+ monocytes were gated for the absence or presence of GFP-fluorescence and analyzed for their percentage of hypodiploid DNA (Fig. 1D; n = 14, * p<0.05).

We verified cell death via apoptosis by three methods to monitor distinct stages (Fig. 1A–C). Four hours p.i. 42.7±7.9% of monocytes exhibited PS at the outer leaflet (CD14+/Annexin V+), 35.05±8.1% exhibited DNA strand breaks (CD14+/TUNEL+) and 52.67±6.3% were found hypodiploid. Blockage of bacterial uptake by Cytochalasin D (CytoD) not only abolished phagocytosis with a decrease of the phagocytosis index to 6.0±3.7% (p<0.05 vs. bacteria only; Fig. S2A), as previously described [10]. Apoptosis rate, quantified by percentage of hypodiploid nuclei, was diminished, however still occurred in 22.7+11.1% (Fig. 1C). Therefore we specified, whether only those monocytes would become apoptotic, that had bound or ingested *E. coli*, or whether monocytes without bacterial contact would as well undergo cell death.

In a first approach we analyzed the CD14+GFP+ and the CD14+GFP- subpopulations and quantified their hypodiploid fractions (Fig. 1D; gating strategies are shown in Fig. S2). Four hours p.i. 16.7±4.6% of GFP-positive monocytes were hypodiploid, while the rate of GFP-negative monocyte apoptosis was even higher (33.5±6%, Fig. 1D). These results suggested, that monocyte apoptosis takes place rapidly, and that it occurs in monocytes, which are not actively taking part in phagocytosis of bacteria.

Lymphocytes, identified by the typical location in the forward- vs. side-scatter diagram, did not exhibited signs of apoptosis during this time interval by any of the three methods described, as shown for hypdiploid DNA (Fig. S1).

### Monocyte Apoptosis Occurs without Transport of Bacteria to the Phagolysosome

Infection with *E. coli*-GFP neither allowed to distinguish between bacterial binding to the cell membrane and transfer to the phagolysosome, a definite sign of engulfment to the intracellular compartment, since both conditions appeared GFP-positive, nor to discriminate between monocytes without contact to bacteria, and monocytes that had already degraded bacteria in the phagolysosome, since both appear GFP-negative.

**Figure 2:**
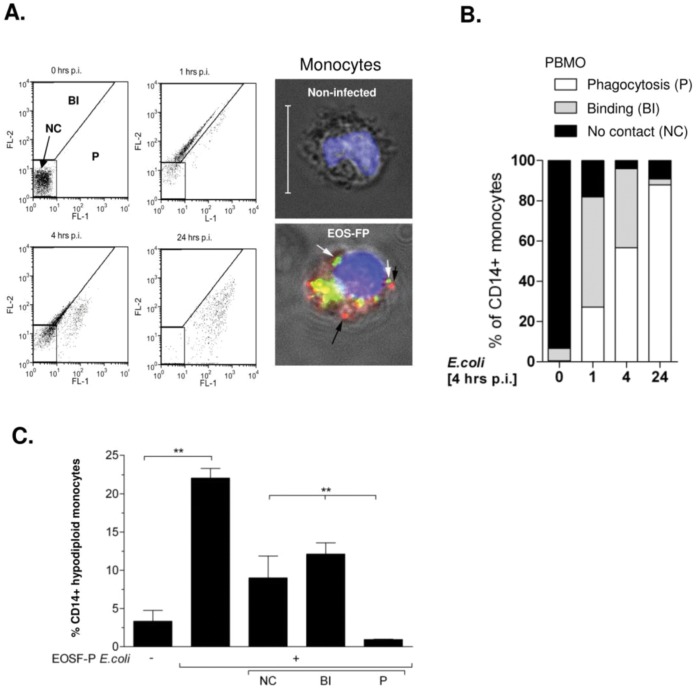
Transport to the phagolysosome is not obligatory for monocyte apoptosis. The gating strategy and nomenclature is shown by dot-plot analysis of monocytes, infected with EOS-FP *E.coli* for the indicated time intervals (A). Bound bacteria exhibit a red fluorescence, in the FL-2 channel (red bacteria in the micrograph, gated BI). Bacteria, transported to the phagolysosome, exhibit a green fluorescence in the FL-1 channel (green bacteria in the micrograph, gate P). Monocytes without bacterial contact were gated in NC. The chart (B) summarizes the proceeding the phagocytic process within the indicated time intervals. Details are given in the supplement. (C): Assessment of monocyte apoptosis by hypodiploid DNA content in total (second column) and in the three subfractions described (n = 7; ** = p<0.001).

Therefore we infected monocytes with *E. coli*-EOS-FP to observe the phagolysosomal transfer by a pH-dependent switch of the EOS-FP fluorophore from a yellow into the green emission spectrum within the acidified lysosome [15]. In this compartment, even degraded bacteria emit the green fluorochrome for up to 24 hours [15]. We infected PBMC for various intervals and discriminated three CD14+ subpopulations as described [15]: Monocytes, which had no contact (gate NC), had bound or ingested (gate BI), or had transferred *E. coli* to their phagolysosome (gate P, Fig. 2A). Fig. 2A shows the development of the subpopulations one, four, and 24 hours p.i. (Table S1). The percentage of monocytes, binding or ingesting *E. coli*-EOS-FP (BI), increased until 4 hours p.i. Within this interval, more than 50% had transferred *E. coli*-EOS-FP to their phagolysosome (P), increasing to 87.9±4.9% 24 hours p.i. Accordingly, monocytes which had bound or ingested (BI) or no contact (NC) decreased.

Comparing these results to the percentage of GFP-positive monocytes after infection with *E. coli*-GFP (66.8±13.1%,), the percentages in the BI- and P-gate in the *E. coli*-EOS-FP model (81.9±10.9%; Fig. 2B, Table S1) made up almost an equivalent fraction.

Infection with *E. coli*-EOS-FP induced monocyte apoptosis, as detected by hypodiploid nuclei (Fig. 2C) 4 hours p.i. Their total apoptosis rate (gates NC+BI+P) was 22.1±1.4% (p<0.05 vs. control, Fig. 2C second column). Their percentage in the NC-gate was 8.5±3%, and in the BI-gate 12.3±1.5% (Fig. 2C third, fourth columns); in both significantly higher compared to the P-subpopulation (1.3±0.2%; p<0.05). These results suggested that transport to the phagolysosome, and/or binding or ingestion was not obligatory for apoptotic cell death.

### Bystander Apoptosis Occurs without Cell-cell Contact

**Figure 3:**
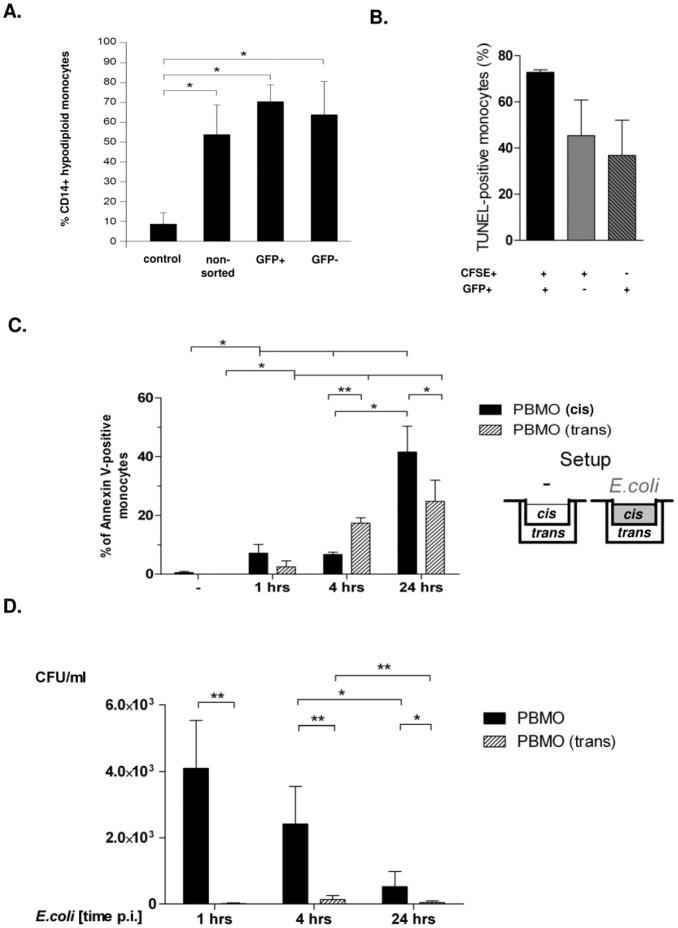
Bystander apoptosis occurs without cellular contact to the phagocyting monocyte. Monocytes were infected with *E. coli*-GFP and GFP –positive and -negative cells were separated by FACS sorting. Apoptosis was assessed 24 hours p.i. by quantification of hypodiploid DNA (Fig. 3A; n = 3, *p<0.05). Monocytes were infected with *E.coli*-GFP for one hour and free bacteria were removed. CFSE labelled monocytes of the same donor were co-incubated for 4 hours in a concentration of 10∶1. Un-infected, unlabelled monocytes served as controls. Apoptosis was assessed 4 hours p.i. in GFP+ and CFSE+ monocytes (B; n = 5). Monocytes were infected with *E. coli*-GFP as described (i.e. 1 hour w/o antibiotics and 3–21 hours with gentamycin) in a transwell setup for the time intervals indicated (“*cis*” chamber, C, sketch to the right); non-infected cells served as controls. Monocytes from the same donor were co-cultivated in compartments, separated by teflon membranes (“*trans*” chamber). Apoptosis was detected by assessment of hypodiploid DNA-content (C, n = 5; * p<0.05, ** = p<0.001). In the same experimental setup, a CFU-assay was performed by lysis of cells (Fig. 3D, n = 5; * p<0.05, ** p<0.001).

To address, whether induction of monocyte apoptosis without bacterial contact requires intercellular contact to phagocyting monocytes, three experimental approaches were chosen: Cell sorting, co-culture of viable, CFSE-labelled non-infected monocytes, and transwell experiments (Fig. 3).

CD14+ monocytes were purified, infected with *E. coli*–GFP for 60 minutes, and GFP-positive were separated from GFP-negative monocytes by cell sorting (Fig. S3A, B). 24 hours p.i. apoptosis was determined by quantification of hypodiploid nuclei. The percentage of apoptotic monocytes in the sorted GFP-positive and GFP-negative population was equivalent (65±17% vs. 72±9%) and not different from the originating unsorted monocyte population (55±15%, Fig. 3A). The apoptosis rate of non-infected purified monocytes was low (8.1±4.9%; Fig. 3A). Prior to cell sorting 30±27% of monocytes were GFP-positive, afterwards GFP-positive monocytes were nearly absent in the GFP-negative fraction (0.6±1%, Fig. S3B).

In a second approach, we co-cultivated previously *E. coli*-GFP infected, repeatedly washed, purified monocytes (infection for 60 minutes; cf. Material and Methods) with non-infected, CFSE-labelled monocytes for 4 hours and assessed apoptosis by detection of hypodiploid nuclei. 72±1.5% of all purified monocytes were hypodiploid (not shown). The percentage of CFSE-labelled, non-infected hypodiploid monocytes (45.3±15.5%) was found equivalent to the percentage of their infected monocytes, reaching 36.8±15.2% (Fig. 3B, Fig. S4).

In a third approach, we infected monocytes with *E. coli*-GFP at one side of a transwell chamber (cis), and co-cultivated non-infected monocytes of the same donor in the adjacent chamber (trans; experimental setup given in Fig. 3C) and analyzed monocyte apoptosis at various time points (Fig. 3C). Within 24 hours p.i., monocyte apoptosis was induced in both, the cis-chamber of infected monocytes (41.6±8.8%), and, to a lesser extent, in the adjacent trans-chamber (24.8±7.2%). Onset of apoptosis, assessed by annexin V staining, was detectable in the trans-chamber already after 4 hours p.i. (17.6±1.9%).

In order to rule out fast bacterial degradation, we documented the number of surviving bacteria in a time course via a colony-forming-unit (CFU) assay (Fig. 3D): The number of phagocyted, alive intracellular bacteria revealed 4×10^4^ bacteria one hour p.i (4096±717 CFU/ml vs. controls; infected with non-proliferative, fixed *E.coli*). Further cultivation decreased the bacterial amount to 2417±567 CFU/ml 4 hours p.i., dropping to 0.5×10^4^ CFU/ml 24 hours p.i. No bacteria were found in the adjacent trans-chamber. These results suggested that soluble factors were able to induce apoptosis in monocytes located at the trans-chamber without physical contact to bacteria or infected cells.

### Soluble TNF-α Contributes to Monocyte Apoptosis

**Figure 4:**
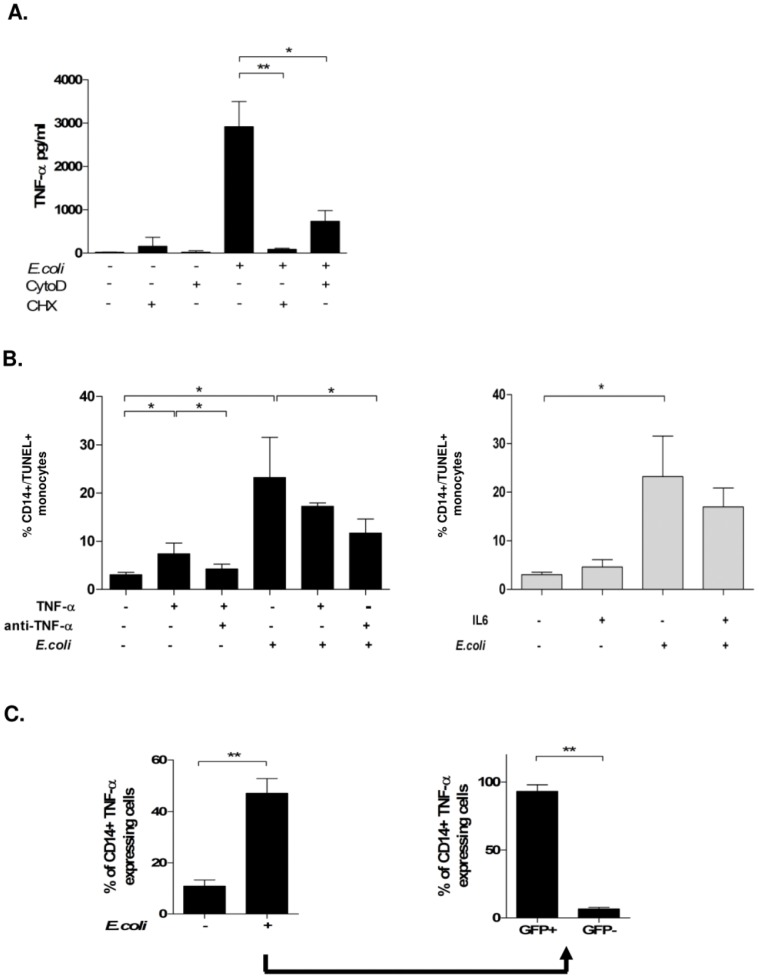
Infection with *E. coli* induces TNF-α production and monocyte apoptosis in phagocyting and non-phagocyting cells. Monocytes pre-incubated with Cyto D or CHX were infected with *E. coli*-GFP for 4 hours and TNF-α production was detected in culture supernatants by ELISA (A; n = 8, * p<0.05.) In the same experimental setup, monocyte apoptosis was quantified by TUNEL-staining after treatment with TNF-α neutralizing anti-TNF-α antibody, or *E. coli*, or combinations (B, left;; n = 9, * p<0.05), or IL-6 (B, right; n = 4, * p<0.05). In the same setup, intracellular TNF-α production of monocytes was analyzed by FACS-stain (C, left) and gated for GFP-positivity (C; n = 5, * p<0.05, ** p<0.001).

Infection with *E. coli* induced a robust TNF-α production, peaking 4 hours p.i. (Figure 4A). The percentage of TNF-α producing monocytes increased after infection from 11.5± % to 47.2±5.6% 4 hours p.i. (Fig. 4C, left panel). Blockage of protein *de-novo* synthesis by cycloheximide (CHX) nearly abrogated the TNF-α production. The latter also was dependent on a functional cytoskeleton, since the addition of CytoD reduced TNF-α secretion to 25%, suggesting that an intact actin-cytoskeleton was required for TNF-α secretion (Fig. 4A).

To underline that TNF-α secretion was not an epiphenomenon with regard to monocyte apoptosis, we also investigated the reaction in response to the pro-inflammatory cytokine IL-6, the secretion of which is well documented in clinical sepsis. IL-6 as well was produced in sizable amounts, reaching 1600.9±620.8 pg/mL 4 hours p.i. and did not influence bacterial phagocytosis (Table S2) Whereas addition of TNF-α induced apoptosis in uninfected monocytes (Fig. 4B left)., supplementation of external IL-6 did not induce apoptosis (Fig. 4B, right).

TNF-α-mediated apoptosis could be inhibited to 50% by an anti-TNF-α antibody (Fig. 4B, left). TNF-α did not enhance apoptosis in *E. coli*-infected monocytes, while phagocytosis was not affected (Table S2). Notably, anti-TNF-α mAb reduced apoptosis in infected monocytes by 65% as analyzed by DNA strand breaks (Fig. 4B) and quantification of hypodiploid nuclei (Table S3).

### TNF-α is Mainly Produced by Phagocyting Monocytes While its Production in Bystander Cells may Protect them from Apoptosis

**Figure 5:**
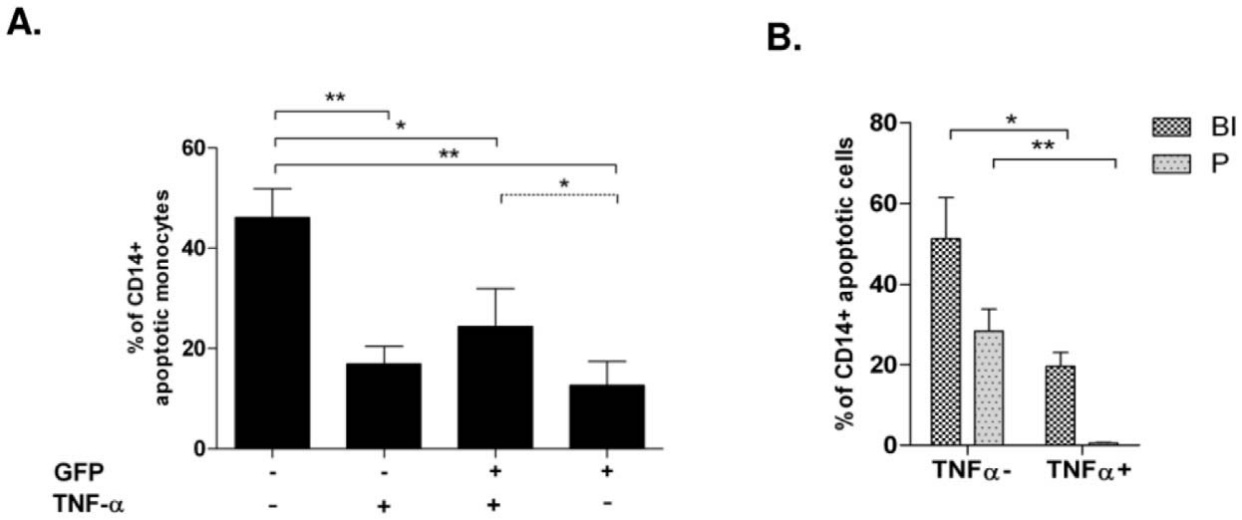
Monocyte apoptosis with regard to bacterial phagocytosis and TNF-α production. Monocytes were infected with *E.coli*-GFP for four hours and free bacteria were removed. Four hours p.i. hypodiploid DNA content was quantified and analyzed for absence or presence of intracellular viable bacteria (GFP-negative or positive, respectively), and TNF-α content (A; n = 7, * p<0.05, ** p<0.001). Monocytes were infected with *E.coli*-EOS-FP for one hour and free bacteria were removed. 4 hours p.i. we quantified hypodiploid DNA content in TNF-α -positive and –negative monocytes in the BI- and P-gate (B; n = 5, * p<0.05, ** p<0.001).

To further identify the source of TNF-α, we analyzed GFP-positive and GFP-negative monocytes and found that 92.9±2.4% of GFP-positive cells produced TNF-α, while only 7.1±0.9% of GFP-negative were positive for TNF-α (Fig. 4C, right panel). Thus we wondered, whether TNF-α production would protect from apoptosis and quantified hypodiploid nuclei in monocytes four hours p.i. (Fig. 5A): GFP-negative monocytes, which had no intracellular TNF-α content, were most prone to apoptosis (46.1±5.8%). GFP-negative, TNF-α-positive monocytes had a lower apoptosis rate (16.9±3.5%, p<0.001). As shown before (Fig. 1B), GFP-negative monocytes became more apoptotic than GFP-positive (58.8±5.2%vs. 41.3±5.5%, columns one and two vs. three and and four). In the latter population, TNF-α production had no additional protective effect, since more GFP-positive, TNF-α-producing monocytes became apoptotic than GFP-positive cells without TNF-α (Fig. 5A, columns three and four).

To discriminate between binding and phagocytosis in view of TNF-α production and apoptosis induction, monocytes were infected with *E.coli-EOS-FP* (Fig. 5B): In *E. coli* binding monocytes (gate BI), which did not produce TNF-α, the apoptosis rate was significantly higher compared monocytes, which already had processed the bacteria to the phagolysosome (gate P) and did not produce TNF-α.

Taken together, these results indicate, that direct bacterial contact may trigger TNF-α production in phagocyting monocytes, but its production in non-phagocyting monocytes may mediate protection from apoptosis. Together with previous results (Fig. 2) the data further indicate, that bacterial transport to the phagolysosome may trigger anti-apoptotic signals independent from TNF-α production.

### TNFR1 Expression is Down-modulated after Infection, Internalized and Involved in Pro-apoptotic Signalling

**Figure 6:**
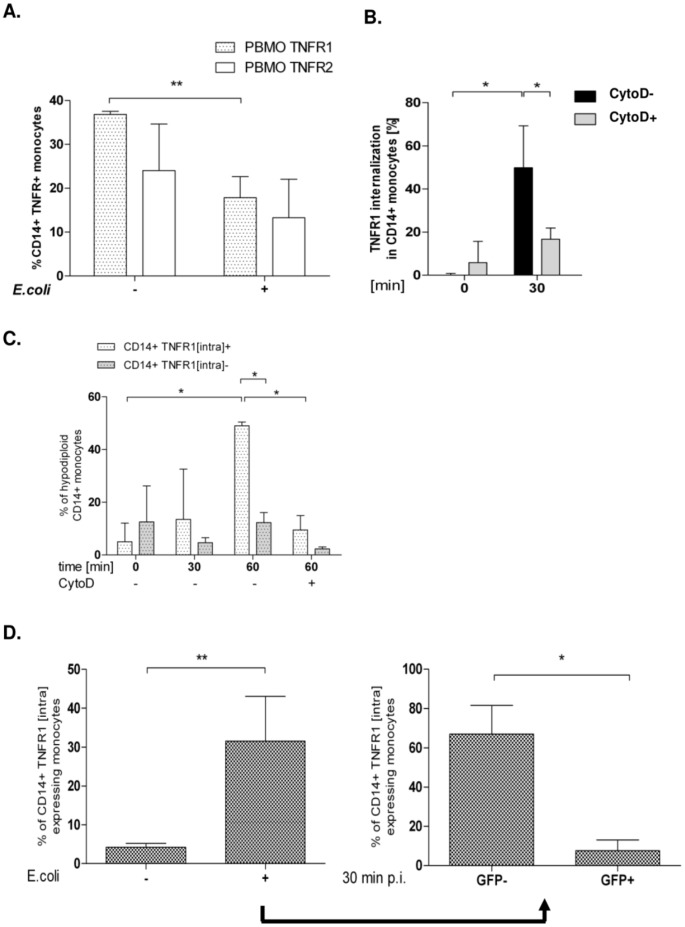
TNFR1 is internalized after incubation with TNF-α and after infection with *E. coli* and leads to monocyte apoptosis. Monocytes were infected with *E.coli*-GFP for one hour and free bacteria were removed. 4 hours p.i. TNFR1 and -2 expression were analyzed by FACS (A; n = 9, **p<0.01). Analysis of TNFR1 internalization following TNF-α administration after 30 minutes; one group received CytoD (B; n = 7, p<0.05). In the described experimental setup, monocyte apoptosis was analyzed via Vybrant DNA-content and distinguished by internalization of TNFR1 (C, * p<0.05, ** = p<0.001). One group was cultivated with CytoD. TNFR1 internalization 30 min p.i. Internalization was quantified for GFP-negative and –positive monocytes (D; n = 5,* p<0.05, ** p<0.001.

Since TNFR1 signalling has been associated with apoptosis-induction, we analyzed TNFR1 and -2 expression after infection. In contrast to TNFR2, TNFR1 expression was significantly down-regulated on monocytes four hours after infection with *E. coli*-GFP (Fig. 6A). Intracellular staining revealed, that TNFR1 was internalized rapidly, within 30 minutes after addition of external TNF-α to uninfected monocytes (Fig. 6B). Monocytes, which had internalized TNFR1 as a consequence of TNF-α addition, were found significantly more apoptotic than monocytes with their receptors still on the surface (Fig. 6C). No apoptosis was detected, when only labelled anti-TNF-α mAb was given (not shown).

Addition of CytoD not only reduced TNFR1 internalization by 65% (Fig. 6B, column three vs. four) but further led to a significant inhibition of TNF-α-mediated apoptosis after 60 minutes (Fig. 6C, columns five and six vs. seven and eight; p<0.05), pointing to a pivotal role of TNFR1 internalization in TNF-α mediated monocyte apoptosis.

Having shown that monocytes, which had internalized TNFR1 in response to external TNF-α became apoptotic (Fig. 6B,C), that TNF-α was produced (Fig. 4A), and TNFR1 was down-regulated during infection with *E. coli* (Fig. 6A), we analyzed TNFR1-internalization in our *E. coli*-GFP model: GFP-negative monocytes internalized TNFR1 significantly more than GFP-positive cells 30 min p.i. (Fig. 6D). Together with the observation, that a great number of GFP-negative monocytes became apoptotic (Fig. 1D), these data suggest that TNFR1 internalization may be crucial in the TNF-α mediated cell death of bystander monocytes.

## Discussion

In this study we demonstrate that *in vitro* infection of monocytes with *E. coli* resulted in apoptotic cell death of the phagocyting cell and of bystander monocytes, not taking part in phagocytosis, that TNF-α played a crucial role in apoptosis induction and that different mechanisms were involved in apoptosis of phagocyting and bystander cells. Dissecting the cellular events during phagocytosis and apoptosis revealed, that apoptosis of phagocyting cells (1) was independent of transport of bacteria to the phagolysosome, (2) was accompanied by significant TNF-α production and that (3) active phagocytosis and TNF-α production partly protected from apoptosis compared to bystander cells. Apoptosis of bystander monocytes occurred (4) mainly independent of contact to phagocyting cells, (4) required protein *de novo* synthesis and (5) was induced by TNF-α. (6) TNF-α mediated TFNR1 internalization was crucial for apoptosis induction.

Apoptosis-inducing signals in monocytes during their interaction with bacterial pathogens mostly have been linked to direct binding and/or the process of phagocytosis, thus leading to the term of “phagocytosis induced cell death” - PICD. PICD, i.e. cell death of the phagocyting cell, has been described to be induced by different bacterial species, independent from bacterial toxins, in various phagocyting cells, mainly granulocytes and monocytes. Independently, it has been shown for a considerable time that phagocyting monocytes are capable to induce apoptotis in bystander cells. We show here, that both processes occur in parallel and that PICD as well as bystander cell death was induced rather fast, within four hours.

With respect to **PICD**, the inhibition of actin polymerisation by CytoD reduced bacterial phagocytosis (Fig. S1A), and resulted in a reduced apoptosis rate (Fig. 1C), indicating that active phagocytosis may stimulate cell death. Indeed, the actin cytoskeleton has been shown to play a regulating role in several signaling pathways, including apoptosis induction and actin depolymerisation was linked to both, increase and inhibition of apoptosis. Our results furthermore show, that inhibition of actin polymerization hampered TNF-α secretion, a factor crucial for apoptosis induction (Fig. 4A) and internalization of TNFR1 (Fig. 6). In spite of decreased phagocytosis, a considerable apoptosis rate was still detectable after preincubation with CytoD (Fig. 1C), suggesting that either the small fraction of phagocyting monocytes were potent enough to induce apoptosis and/or that apoptosis appears independently of actin dependent phagocytosis.

The observation, that CytoD on the one hand decreased phagocytosis (Fig. S1A), but not the contact between bacteria and monocytes, but on the other hand no increase in apoptotic monocytes was detected (Fig. 1C), could possibly be attributed to the simultaneous lack of internalized TNFR1 (Fig. 6C).

With regard to the role of the phagocytic progress in PICD, results are conflicting: Whereas phagocytosis and transition to the phagolysosome were found essential for induction of monocyte apoptosis [25], related to maturation and acidification of this compartment with an activation of apoptosis-modulating factors [26], [27], our finding (Fig. 2) confirms results from other infection models using *N. meningitides*, *K. pneumoniae, Y. enterocolitica*, *E. coli* and fungi, where apoptosis was induced independently of phagocytosis [28], [29], [30].

Using EOS-FP labeled *E. coli* for the first time allowed a clearer discrimination between early steps of phagocytosis, i.e. pathogen binding and formation of endosomes and late steps of phagocytosis, i.e. maturation of acidified phagolysosomes (Fig. 2). The results shown provide a rough time scale for intracellular transport of bacterial prey to the phagolysosome and indicate, that acidification might take as long as 24 hours to be accomplished (Table S1). Our results suggest that monocytes with mature phagolysosomes, as seen in the phagocyting fraction of monocytes (P; Fig. 2B), become less apoptotic than monocytes which only bind/ingest bacteria in early endosomes (BI; Fig. 2C) or which have no contact with bacteria (NC, Fig. 2C). This may indicate, that early steps of the phagocytosis process are accompanied by pro-apoptotic signals while the transition of bacteria to the phagolysosome may provide anti-apoptotic signals leading to cell survival.

Some studies found phagocytosis, and transition to the phagolysosome essential for induction of monocytic apoptosis. Bacterial degradation in the phagolysosome was related to maturation and acidification of this compartment, which in turn caused the activation of apoptosis-modulating factors. In contrast to this, in our experimental setup the transition of bacteria to acidified phagosomes was a slow process that took time up to 24 hours. Monocyte apoptosis however was detectable as soon as 4 hours p.i., thus maturation to acidified phagolysosomes was not necessary for apoptosis induction in our experimental system.

With respect to **bystander apoptosis,** this process was detected not only in experiments with mononuclear cells containing mainly T cells apart from monocytes, but also when using isolated monocytes (Fig. 3), and since we could identify TNF-α as a major apoptosis inducing factor, which is mainly produced by monocytes, it is most likely, that bystander apoptosis is mainly induced as “fratricide” by monocytes. Our data indicate, that bystander apoptosis was mainly induced paracrine (Fig. 3), by soluble “death ligands” such as TNF-α (Fig. 4, 5), which were synthesized during the infection process,without the necessity of intercellular contact (Fig. 4A).

TNF-α is known to bind to its receptors in a membrane-bound (mTNF-α) and a soluble form (sTNF-α). Soluble TNF-α activates TNFR1, whereas mTNF-α initiates TNFR2 signalling, resulting in a pro-inflammatory and less apoptosis inducing response [9], [12]. Our findings underline this functional model, since both TNF-α receptors were modulated differently after *E.coli* infection (Fig. 6A): In contrast to TNFR1 surface expression, which was significantly reduced, TNFR2 receptor expression remained almost unaltered.

Experimental data from TNFR1/TNFR2 knock-out models demonstrate an intricate situation. TNFR1−/− and TNFR1+2−/− double knock-out mice showed a prolongued survival in ceacal-ligation puncture experiments and suggest that TNFR1 is the receptor required for pro-apoptotic signaling [31], Secher). Internalization is required for a pro-apoptotic signaling of TNFR1 [12]. Our data, which provide evidence for a quick translocation of TNFR1 to intracellular compartments (Fig. 6), are in line with these observations. Since GFP-negative monocytes internalized TNFR1 to a higher extent, our data suggest that the cell death of non-phagocyting bystander monocytes is initiated by TNFR1 internalization, and that the source of TNF-α triggering this process, is the phagocyting mate (Fig. 4B, Fig. S5).

Both forms of TNF-α can ligate TNFR1 and TNFR2 at the plasma-membrane thereby activating the expression of pro-inflammatory genes and enhancing its own production via *de-novo* synthesis after activation of the NF-kappaB pathway [9], [11], [21]. We showed that *de-novo* synthesis was obligatory, since its inhibition by CHX reduced the secretion of TNF-α threefold (Fig. 4A).

IL-6, another cytokine of the pro-inflammatory pathway, could contribute as well to bystander-apoptosis, since IL-6 may lead to NF-kappaB driven production of reactive oxygen species, resulting in activation of the intrinsic apoptotic pathway [28], [32]. In contrast to TNF-α we found IL-6 neither to induce apoptosis in the concentration range used, nor to enhance PICD after infection with E. coli (Fig. 4B). IL-6 is suggested to be a less potent activator of the pro-apoptotic NF-kappaB pathway [33], which is in line with our observation.

The analysis of a relation between intracellular TNF-α content and monocyte apoptosis (Fig. 4C, 5), revealed TNF-α production mainly to occur in phagocyting cells (Fig. 4C). We found different subpopulations with regard to apoptosis: Both, GFP-positive and GFP-negative TNF-α producing subpopulations were found to be less sensitive towards apoptosis than the GFP-negative, non-TNF-α producing monocytes (Fig. 5A, columns one to three). We found apoptosis also diminished in monocytes with advanced phagocytosis with no TNF-α production (Fig. 5A, B), which again was reduced, when the bacteria already had reached the phagolysosome (Fig. 5A fourth column; 5B second column). This finding indicates protective mechanisms against TNF-α-mediated bystander apoptosis by endogenous TNF-α production. Our experimental setup, though cannot rule out, that TNF-α-negative monocytes had already secreted the majority of TNF-α, or that other mediators, e.g. CD95L, are involved.

Thus, it appears that in the course of bystander apoptosis, the dynamics of TNF-α localization and TNFR1 internalization play a critical role in balancing internalization-dependent apoptotic and internalization-independent non-apoptotic pathways to elicit cell death and other functions, respectively.

The balance of pro- and anti-inflammatory mediators defines the progression and severity of sepsis. An overproduction of endogenous proinflammatory mediators, including cytokines, synergistically interact to mediate hypotension, multiple organ failure, and death. Progression from sepsis to septic shock coincides with the increase in circulating levels of proinflammatory cytokines such as TNF-α, interferon gamma, IL-1, and IL-6 [34] In animal models, neutralizing antibodies to TNF prevented death when administered before or concurrent with lethal doses of live *E. coli* [35]. In this context, apoptosis of immune cells may play opposing roles for the clinical course of sepsis. While lymphocyte depletion by apoptosis was correlated with poor outcome, prolonged survival of monocytes worsened the course. So, at present it is far from clear which consequences result from loss of innate immune effector cells during infection. Although apoptosis may be an appropriate part of a host response, its tight regulation and its temporal relationship with phagocytosis are critical in regulating both bacterial elimination and controlled inflammation. Modifying monocyte bystander apoptosis in sepsis, targeting the TNFR1-expression may be a therapeutic tool in future.

## Materials and Methods

### Patients

The study protocol was approved by the Ethics Committees of Aachen University Hospital (Permission No: EK150/09, Oct. 6, 2009, signed by Profs G. Schmalzing and U. Buell, respectively). All participants involved in our study gave written consent to use their blood samples serving as controls.

### Reagents

Antibodies to CD14 (MFP9; MEM18), TNFR1 (55R–286) and Ig-matched controls (IgG1, IgG2b) were from BD Biosciences and Immunotools (Heidelberg, Germany and Friesoythe, Germany, respectively). The secondary anti-mouse-PB antibody (F`ab fragment) was from Invitrogen (Darmstadt, Germany). Diamidino-2-phenylindole-dihydrochloride (DAPI) was from Merck (Darmstadt, Germany). Propidium iodide (PI), isopropyl-β-D-thiogalactopyranoside (IPTG) and antibiotics were purchased from Sigma (Munich, Germany). Vybrant was obtained from Invitrogen (Paisley, UK). The TUNEL apoptosis kit was purchased from Roche (Mannheim, Germany). Actin-dependent uptake of bacteria was blocked by cytochalasin D (Cyto D, Sigma, Munich, Germany; 10 µg/ml) 30 minutes prior to infection. Protein *de-novo* synthesis was analyzed by cycloheximide (CHX; Sigma) at a final concentration of 100 µg/ml 60 minutes prior to infection. To maintain the abrogation of the protein *de-novo* synthesis, a CHX boost was applied after FCS purification as described.

The same procedure was chosen for the administration of an anti-TNF-α antibody (anti-TNF-αmAb), a chimeric molecule combining the ligand-binding domain of the TNF-receptor 2 and the Fc-domain of human IgG1 (ENBREL, Pfizer–Wyeth, Hamburg, Germany, final concentration 1 µg/ml). TNF-α was purchased from Ebiosciences (Ebiosciences-Natutec, Frankfurt, Germany), aliquotted freshly after dilution in PBS and used in apoptosis induction assays in final concentrations of 5 ng/ml (titrated from 0.2, 0.4, 0.8 ng/ml). IL-6 was purchased from Sigma (Sigma, Munich, Germany) and added at a final concentration of 5 ng/ml to cultured cells. Antibodies to the TNF-receptors 1 (MABTNFR1-B1) and 2 (hTNFR-M1) were purchased from Becton Dickinson (BD Biosciences, MountainView, CA, USA). Staining was performed according to the manufacturer`s recommendations. After removal of primary antibodies, a secondary appropriate fluorochrome-labelled antibody was used. For internalization experiments, monocytes were stained with fluorochrome labelled anti-CD14 mAb on ice, washed extensively followed by incubation with anti-TNFR1 mAb for additional 20 min on ice. Thereafter, monocytes were infected with *E.coli*-GFP or TNF-α as described. After 30 min and 2 hours samples were taken and incubated on ice with a secondary anti-mouse antibody. Afterwards, Monocytes were fixed and permeabilized by using the Perm−/Wash solution (BD Biosciences).

### Bacteria

#### *E. coli*-GFP

*E. coli* DH5α, an encapsulated K12 laboratory strain, carrying the green fluorescent protein (*gfp*)-mut2 gene (*E. coli*-GFP) was a generous gift from Prof. Dr. Dehio (University of Basel, Switzerland) and was used for phagocytosis as previously described. Bacteria were freshly grown in Lennox-L-Broth-medium (Invitrogen) until early logarithmic growth, resuspended in phosphate-buffered-saline (PBS) and used immediately. Infection was performed at a multiplicity of infection (MOI) of 25∶1 which was achieved by dilution with PBS. The phagocytosis assays were performed as described [23]. The phagocytosis index (CD14+GFP+ monocytes : CD14+ monocytes) was analyzed by flow cytometry.

#### *E. coli*-EOS-FP

Fixed, EOS-FP expressing *E.coli* (*E. coli*-EOS-FP, Eos-fluorescent protein, UV-induced fluorescent protein of *Lobophyllia hemprichii*) were a generous gift from Prof. Dr. Schneider (University Hospital of Ulm, Germany). Phagocytosis assays were performed by co-cultivating 1×10^6^ mononuclear cells with 2.5×10^7^ *pfu* (plaque forming units) for times indicated. EOS-FP is a fluorochrome, which exhibits an emission shift from the yellow to green spectrum upon acidification and is suitable for monitoring bacterial transfer to the phagolysosomal compartment. Monocytes were divided into three gates (no bacterial contact (NC); binding/ingesting (BI); transfer to phagolysosome (P), as described [36], [37]. To adjust the gates for the phagocytosis assay, non-phagocyting lymphocytes served as negative controls exhibiting no shift of fluorescence emission (not shown). Monocytes binding *E. coli*- EOS-FP were identified by a (FL-2)PE/(FL-1)FITC emission ratio of 1 (cells in gate BI) as described. Phagocytic activity was defined as the percentage of EOS-FP positive monocytes exhibiting a (FL-2)PE/(FL-1)FITC emission ratio <1 (cells in gate P).

### Mononuclear Cell Cultures

Peripheral blood mononuclear cells (PBMC) were isolated by density gradient centrifugation on Ficoll cushions (Amersham, Freiburg, Germany) as described previously. Washed cells were resuspended in VLE RPMI-1640 (Biochrom, Berlin, Germany). For analysis of post-phagocytic reactions, cells were counted in an ultraplane Neubauer hemocytometer, placed at 2×10^6^ cells/ml in flat bottom 24 well cell culture plates (Costar, Bodenheim, Germany) containing 10% heat-inactivated fetal calf serum (FCS, Biochrom) and gentamycin (Sigma; 200 µg/ml) and incubated at 37°C.

### Purification of Monocytes

Monocytes were separated by negative selection using magnetic cell sorting (MACS) monocyte isolation kit II (Miltenyi Biotec) according to the manufacturer’s instructions. The purity of the resulting population was >92% CD14 positive cells as detected by FACS.

Alternatively, 1 ml of a mononuclear cell suspension (5×10^7^ cells) was loaded on top of a hyperosmotic Percoll gradient (48.5% v/v Percoll, 0.16 M NaCl) and centrifuged for 15 min at 580×g without break. Cells were collected from the interphase, washed two times in culture medium and were resuspended in 1 ml cell culture medium. Then, the suspension is loaded on top of 3 ml of an iso-osmotic Percoll gradient (8.3% v7v Percoll, 30 mM NaCl) and centrifuged again for 1 min at 580×g. After two times of washing the purification was controlled via FACS analysis (monocyte purity >80%) and the enriched monocytes subjected to the appropriate experimental setup.

Internalization assays were performed following a protocol described for TNFR1 overexpressing cells [12]. In brief TNFR1 antibody was added to monocytes for 20 min on ice. After 2 times of washing with ice-cold PBS supplemented with FCS (10% v/v) TNF-α was administered and monocytes incubated for the indicated time intervals at standard cultivation conditions. Internalization was stopped by fixation on ice as described above. TNFR1 on the plasma membrane was visualized by addition of anti-mouse-FITC secondary antibody. After permeabilization (see above) internalized TNFR1 was visualized by anti-mouse-APC antibodies. Internalized TNFR1 was calculated by subtraction of TNFR1-APC signal from the TNFR1-FITC signal.

### CFSE-Labelling

Carboxyfluorescein-succinimidyl-ester (CFSE) labelling was performed as described [38]. The procedure and our gating strategy are shown in Fig. S4.

### Flow Cytometry

A daily calibrated FACS-Canto flow cytometer (Becton Dickinson, MountainView, CA) was used to perform phenotypic analysis. To prevent nonspecific binding, cells were incubated with 10% fetal calf serum on ice for 10 minutes before staining with pacific-blue (PB)-, fluorescein-isothiocyanate (FITC)-, phycoerythrin (PE)-, allophycocyanin (APC)-, or isotype-specific immunoglobulin-labelled monoclonal antibodies for 20 minutes over ice in the dark. Monocytes were gated by forward (FSC), side scatter (SSC), and CD14 expression.

### Annexin-V Assay

Phosphatidyl-serine (PS) binding Annexin V was provided by Immunotools (Friesoythe, Germany). To 10^6^ cells 2 µl of annexin V solution was added in CaCl_2_ supplemented PBS (2.5 mmol final concentration) for 20 min on ice in the dark.

### TUNEL Assay

Cells were stained with CD14 mAb for 15 minutes at RT before fixation in paraformaldehyde (2% v/v in PBS). Subsequent steps of the terminal-deoxynucleotidyl-transferase dUTP nick-end labelling (TUNEL) assay were performed according to the manufacturer`s recommendations (Roche, Mannheim, Germany). Fixed, permeabilized and DNAse I treated mononuclear cells served as positive controls.

### Hypodiploid Nuclei

DNA fragmentation was assessed according to Nicoletti and previously described [39]. In brief, washed cells were slowly resuspended in 2 ml of −20°C ethanol 70% with continuous vortexing and stored for four hours at −20°C. Cells were washed twice, resuspended in 50 µl PBS containing 13 units RNAse (DNAse free; Sigma) and incubated for 15 minutes at 37°C. 180 µl of propidiumiodide (PI, 70 µg/ml) was added, incubated for 20 minutes and analysis was performed immediately. Alternatively, mononuclear cells were stained with CD14 antibody for 15 minutes at RT to identify monocytes. A fixation with paraformaldehyde (2% v/v in PBS) for 2 hrs at RT replaced the ethanol fixation. Afterwards, cells were permeabilized by incubation in PBS-T (PBS, Triton X-100 0,1% w/v) for 20 minutes at RT, washed twice in PBS, resuspended in PBS-PI (PBS, 70 µg/ml PI and 13 units RNAse) and incubated for 10 minutes at RT before analysis by flow cytometry. Cell-doublets were discriminated by assessment of PI-width/PI-area. Apoptosis detection in experiments utilizing *E. coli*- EOS-FP, was performed by Pacific Blue (UV bandwith emission) Vybrant stain according to the manufacturer’s recommendations (Invitrogen, Paisley, UK). Vybrant, a Hoechst derivative, is transported actively into the nucleus of living cells and replaces the PI dye whose fluorescence emission interferes with EOS-FP. To discriminate necrotic cells from the fraction of hypodiploid cells, Vybrant and PI were used with non-fixed, non-permeabilized cells. Numbers of necrotic cells were always below 10% and were subtracted from the fraction of hypodiploid cells assessed by the Nicoletti staining. The lower percentage of TUNEL-positive cells (Fig. 1B) as compared to AnnexinV (Fig. 1A) and assesment of hypodiploididty (Fig. 1C) may be explained by the higher sensitiveness to fixation and permeabilization. Although we were very careful in the identification (i.e. gating of the subG1 peak representing apoptotic cells, see Fig. S1C) we cannot completely rule out that this technique covers some non-apoptotic (i.e. necrotic, pyroptotic) cells with aberrant DNA content.

### Recovery of *E. coli* after Phagocytosis (Colony Forming Unit- (CFU-) Assay)

Recovery of live *E.coli* after monocytic uptake was performed as described [40]. In brief, mononuclear cells were incubated in PBS-T (PBS supplemented with Triton X-100, 0.05% v/v) for 10 min on ice. After lysis, surviving bacteria were platet on LB-Agar supplemented with the appropriate antibiotics. Plating of the *E.coli* suspension used for infection served as positive control. As a negative control, CFU-assays were conducted with *E.coli* fixed and inactivated prior to infection.

### Fluorescence Activated Cell Sorting

Isolated monocytes were infected with *E. coli* -GFP for 60 minutes as described. Thereafter cells were subjected to cell sorting using a FACS Aria sorter (Becton Dickinson), located at the FACS core facility of Tuebingen University Hospital. 2×10^6^ cells were sorted by discrimination of the GFP-signal as shown in Fig. S1. Sorted cells were cultured for 24 hours as described. Thereafter apoptosis was assessed by quantification of hypodiploid nuclei.

### Microscopy

Mononuclear cells were fixed in paraformaldehyde (2% v/v in PBS) for 20 minutes at RT, washed in PBS, containing 20 ng/l 4, 6-Diamidino-2-phenylindole (DAPI) and mounted with Immu-Mount (Thermo-Scientific, Pittsburgh, USA). Micrographs were taken by a video camera (Visitron systems, Puchheim, Germany) combined with an Axioplan 2 microscope (Zeiss, Germany). Microscopical pictures were analyzed by using the AxioVision (Release 4.8) ZEISS (Germany) software. The scale bar was 0.01 µm.

### Transwell Experiments

For co-cultivation experiments transwell plates (pore diameter of 0.4 µm, purchased from Corning, NY, USA) were used. Cells in the upper chamber were untreated or infected as described above. Cells separated by the Teflon membrane had no contact to bacteria as assessed by microscopical and FACS analysis. A detailed experimental scheme is given in Figure 3.

### ELISA

The TNF-α enzyme-linked immunosorbent assay (ELISA) was purchased from Ebiosciences (Ebiosciences-Natutec, Frankfurt, Germany) and used according to the manufacturer’s recommendations. After stopping the colorimetric procedure, the read-out was executed in a spectra max 340PC ELISA reader (molecular devices, Sunnyvale, CA, USA) with a sensitivity from 4–500 pg/ml.

### Statistical Analysis

Results are expressed as mean +/− standard deviation. Error bars represent standard deviations. Values of p<0.05 were considered significant. Analyzes were done with statistical software (performing student`s t-test and two-way ANOVA adjusted according to Bonferroni-Holm for multiple group comparisons as provided by GraphPad Software Statistical Package, La Jolla, CA 92037 USA).

## Supporting Information

Figure S1 **Apoptosis in CD14-negative cells from adult blood.** Non-infected (top dot plot) and infected (middle dot plot) CD14-negative PBMC were assessed for GFP-fluorescence and apoptosis by hypodiploid nuclei. Histograms show the DNA-content (bottom). Solid black line: Non-infected CD14-negative cells, dotted black line: GFP-/CD14-negative cells, grey solid line: GFP^+^CD14-negative cells (A). (B) summarizes the results (n = 14).
(TIF)

Figure S2 **Gating strategy and** ***in-vitro*** **infection assay.** The experimental flow chart is given in (A), left. Phagocytosis indices of monocytes infected without or with CytoD is shown in A right (n = 14, ** p<0.001). Monocyte gating and discrimination of GFP-positive and –negative monocytes is shown in (B). (C), (D), (E) show representative data on apoptosis of GFP-positive and GFP-negative monocytes (C) Annexin V staining, (D) Nicoletti DNA staining, (E) TUNEL staining.
(TIF)

Figure S3 **FACS-sorting**. The experimental setup is summarized in the sketch (A). CD14+ monocytes were enriched via MACS-sorting prior to infection with *E. coli*-GFP, followed by FACS sorting. Percentage of GFP+ cells in unsorted and sorted cell populations (B, n = 3, * p<0.05).
(TIF)

Figure S4 ***E.coli*** **infection leads to apoptosis in non-infected, purified monocytes.** The sketch gives the experimental setup (A). A representative dot plot analysis (B) gives apoptotic (TUNEL+) purified monocytes after mixing naïve, non-infected (CFSE+) purified monocytes (central dot-plot) and *E.coli-GFP* infected purifed monocytes (right dot-plot).
(TIF)

Figure S5 **Pro- and anti-apoptotic pathways in monocytes after infection**. Drawings summarize possible TNF-α driven signalling mechanisms in adult monocytes.
(TIF)

Table S1 **Uptake and phagocytosis after infection with EOS-FP** ***E.coli.*** Processing of EOS-FP *E.coli* for indicated time intervals. CD14+ monocytes were gated with respect to interaction with EOS-FP *E.coli* (n = 7; p<0.05 *, #, §; p<0.001 **, ##, §§).
(TIF)

Table S2 **Phagocytosis index after infection and treatment with IL-6 or TNF-α.** Phagocytosis indices of CD14+ monocytes infected with E.coli or infected and treated with 50 ng/ml TNF-α or 1 ng/ml IL-6 (n = 5).
(TIF)

Table S3 **aTNF-α mAb impairs monocyte apoptosis.** Mononuclear cells were infected with *E. coli*-GFP for 240 minutes. One group received anti-TNF-α mAb in parallel to bacterial infection. Apoptosis detection (hypodiploid DNA-content) for all CD14+ monocytes and for the subsets of GFP+/CD14+ and GFP−/CD14+ (n = 5; *p<0.05 non-infected vs. infected, # p<0.05 infected w/o anti-TNF-α vs. infected w anti-TNF-α antibodies).
(TIF)
