# Supplementary figures and images for: Infection–induced Bystander-Apoptosis of Monocytes Is TNF-alpha-mediated
Source: PLoS One. 2013 Jan 17;8(1):e53589. doi: 10.1371/journal.pone.0053589 (PMC3547953; doi:10.1371/journal.pone.0053589)

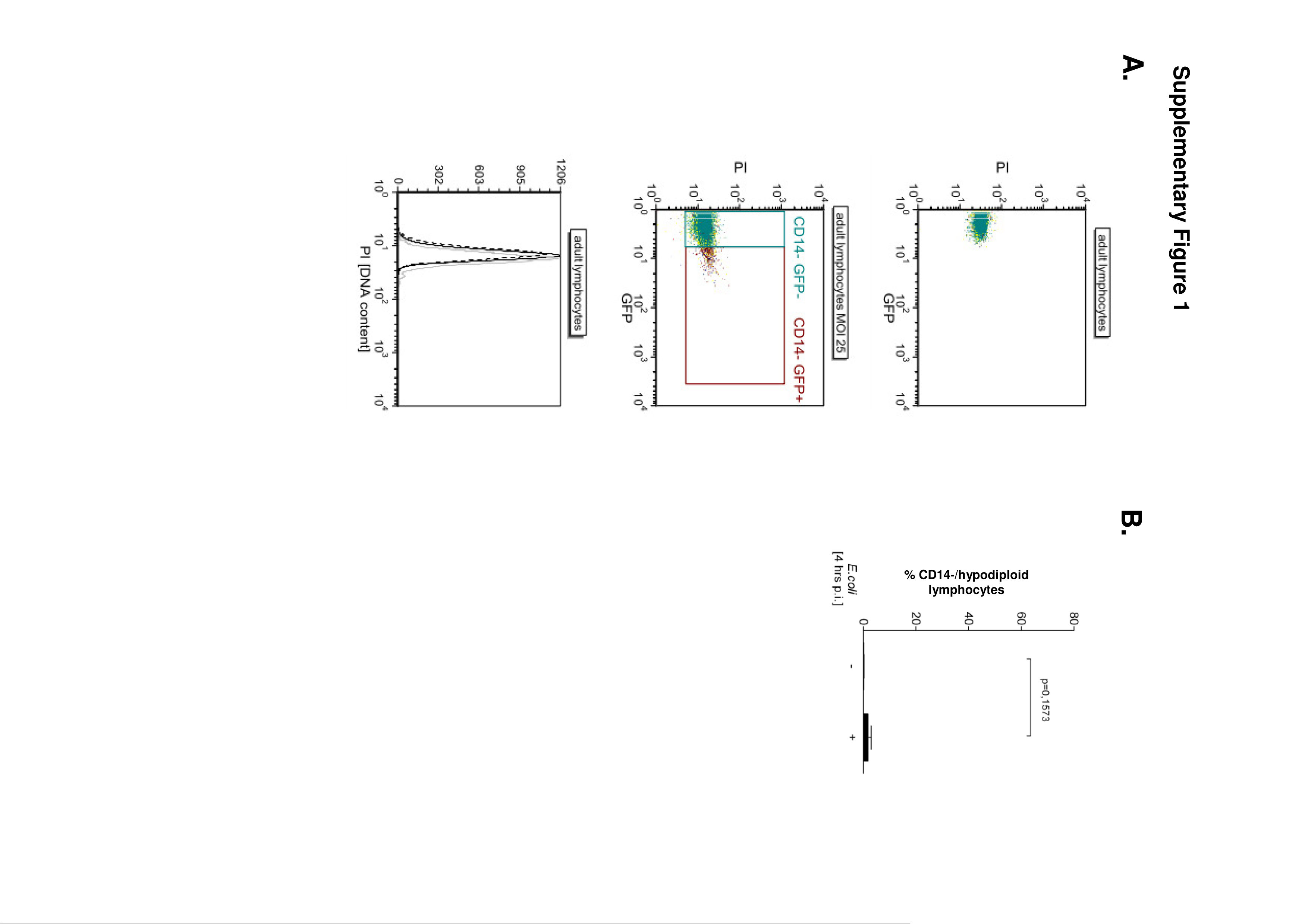

Supplement: Figure S1 — Apoptosis in CD14-negative cells from adult blood. Non-infected (top dot plot) and infected (middle dot plot) CD14-negative PBMC were assessed for GFP-fluorescence and apoptosis by hypodiploid nuclei. Histograms show the DNA-content (bottom). Solid black line: Non-infected CD14-negative cells, dotted black line: GFP-/CD14-negative cells, grey solid line: GFP+CD14-negative cells (A). (B) summarizes the results (n = 14). (TIF) [file pone.0053589.s001.tif]

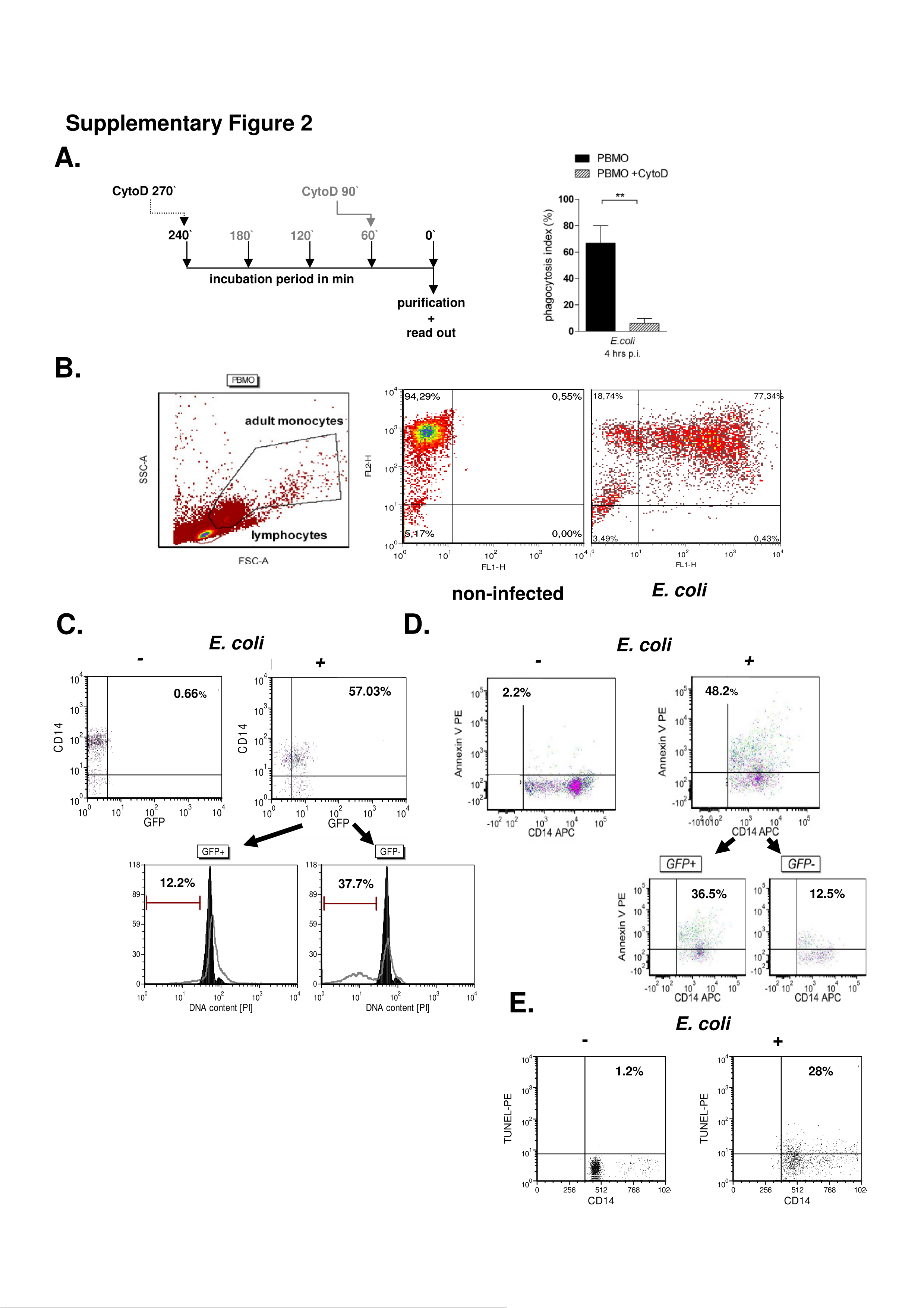

Supplement: Figure S2 — Gating strategy and in-vitro infection assay. The experimental flow chart is given in (A), left. Phagocytosis indices of monocytes infected without or with CytoD is shown in A right (n = 14, ** p<0.001). Monocyte gating and discrimination of GFP-positive and –negative monocytes is shown in (B). (C), (D), (E) show representative data on apoptosis of GFP-positive and GFP-negative monocytes (C) Annexin V staining, (D) Nicoletti DNA staining, (E) TUNEL staining. (TIF) [file pone.0053589.s002.tif]

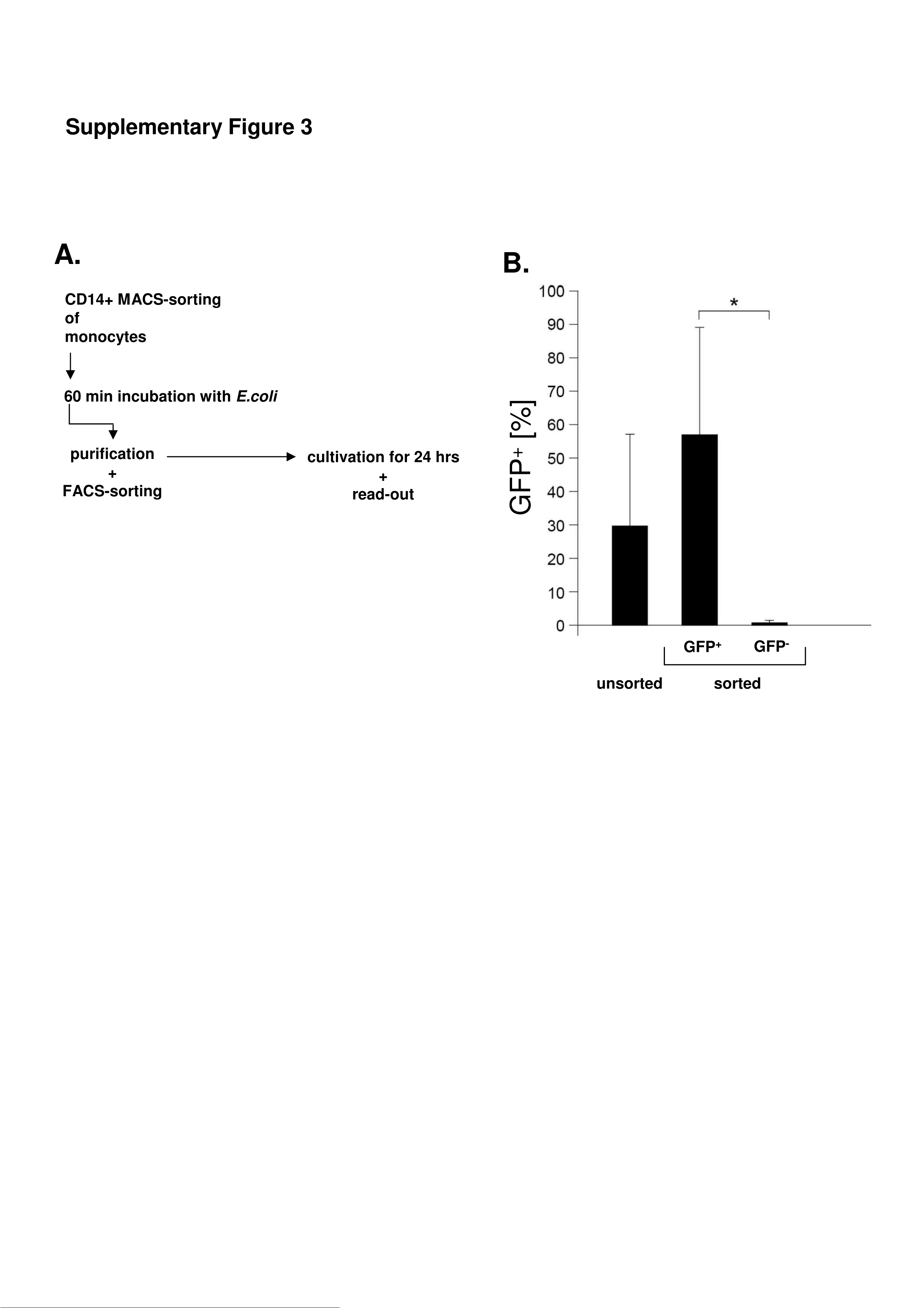

Supplement: Figure S3 — FACS-sorting. The experimental setup is summarized in the sketch (A). CD14+ monocytes were enriched via MACS-sorting prior to infection with E. coli-GFP, followed by FACS sorting. Percentage of GFP+ cells in unsorted and sorted cell populations (B, n = 3, * p<0.05). (TIF) [file pone.0053589.s003.tif]

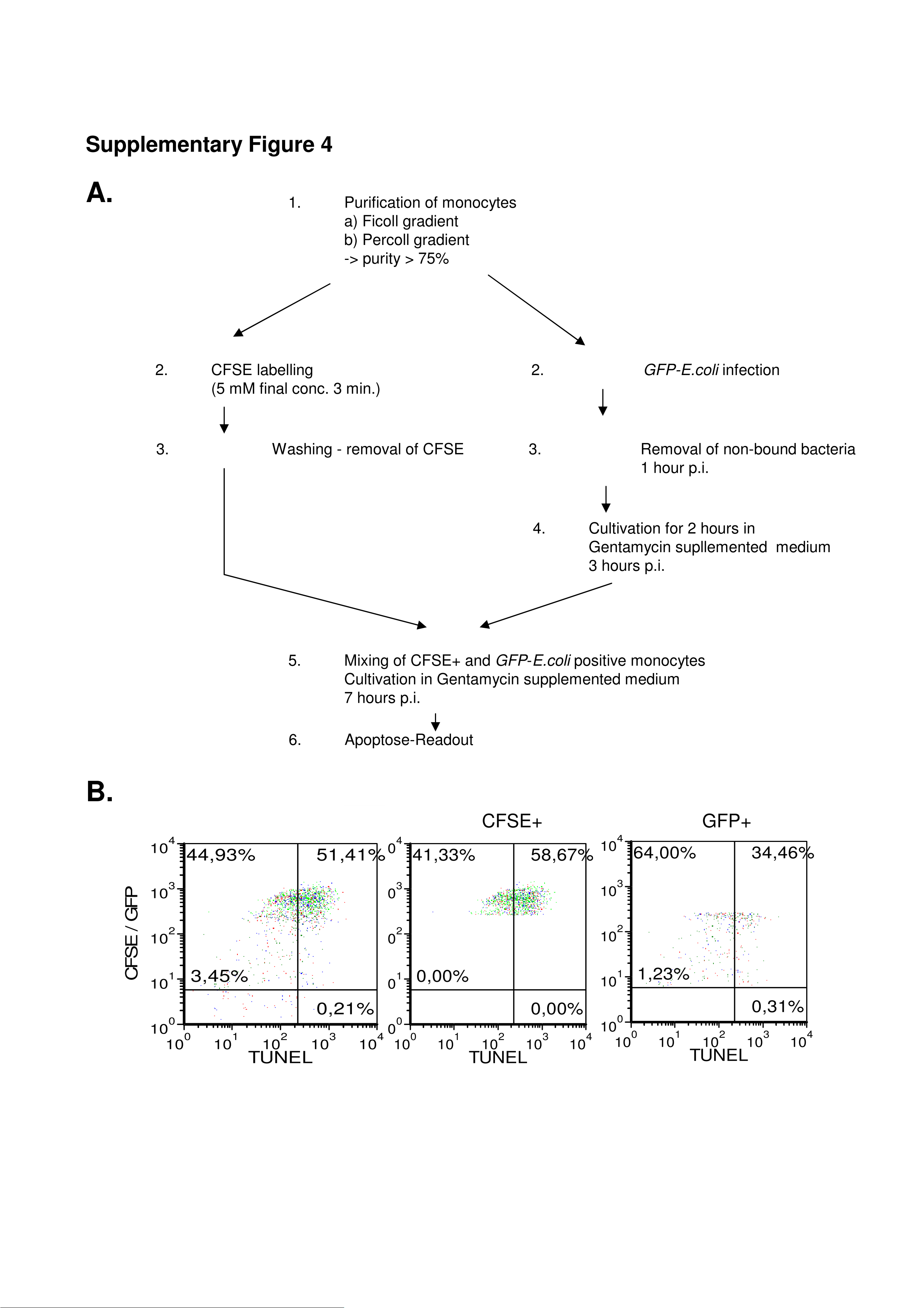

Supplement: Figure S4 — E.coli infection leads to apoptosis in non-infected, purified monocytes. The sketch gives the experimental setup (A). A representative dot plot analysis (B) gives apoptotic (TUNEL+) purified monocytes after mixing naïve, non-infected (CFSE+) purified monocytes (central dot-plot) and E.coli-GFP infected purifed monocytes (right dot-plot). (TIF) [file pone.0053589.s004.tif]

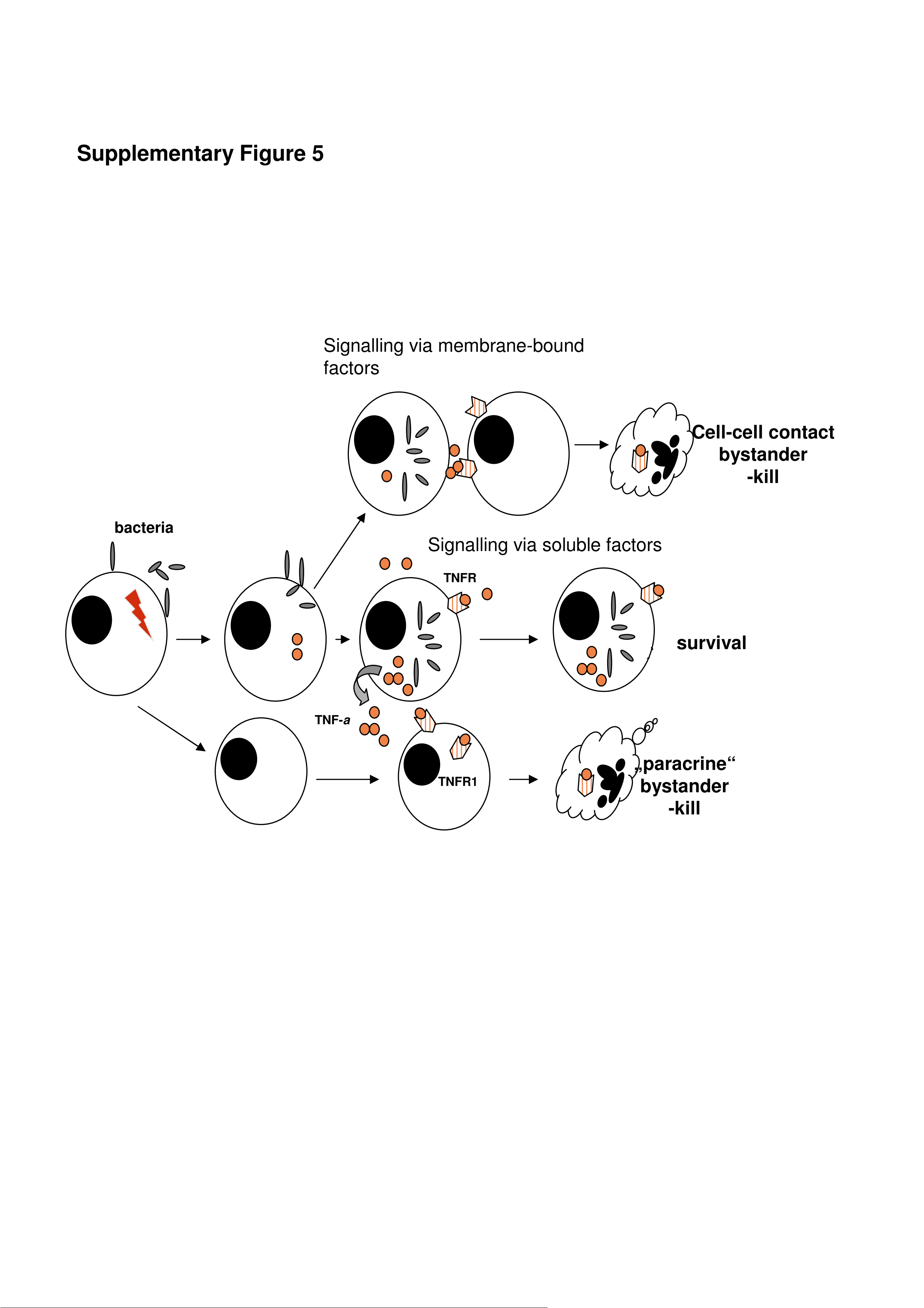

Supplement: Figure S5 — Pro- and anti-apoptotic pathways in monocytes after infection. Drawings summarize possible TNF-α driven signalling mechanisms in adult monocytes. (TIF) [file pone.0053589.s005.tif]

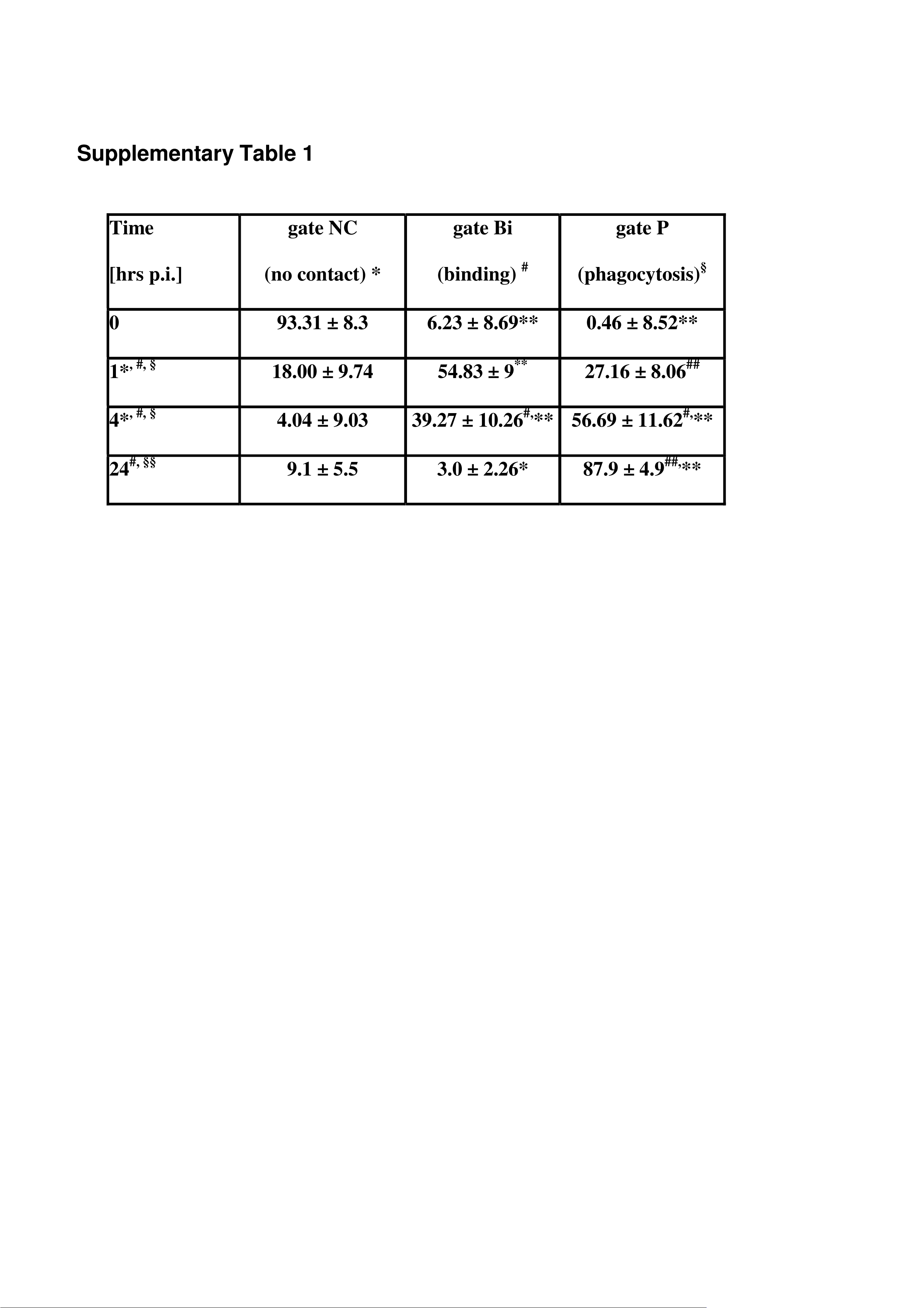

Supplement: Table S1 — Uptake and phagocytosis after infection with EOS-FP E.coli. Processing of EOS-FP E.coli for indicated time intervals. CD14+ monocytes were gated with respect to interaction with EOS-FP E.coli (n = 7; p<0.05 *, #, §; p<0.001 **, ##, §§). (TIF) [file pone.0053589.s006.tif]

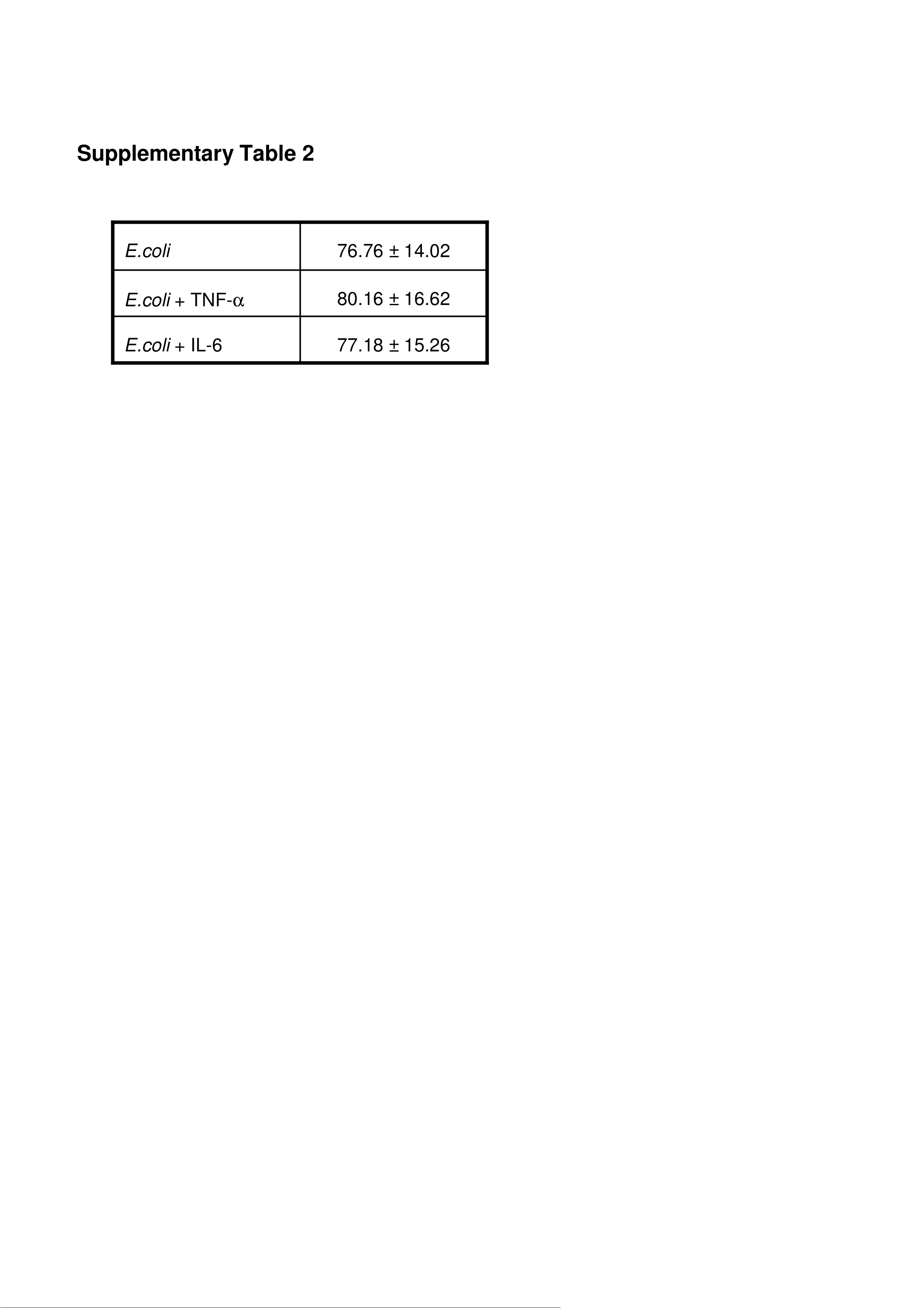

Supplement: Table S2 — Phagocytosis index after infection and treatment with IL-6 or TNF-α. Phagocytosis indices of CD14+ monocytes infected with E.coli or infected and treated with 50 ng/ml TNF-α or 1 ng/ml IL-6 (n = 5). (TIF) [file pone.0053589.s007.tif]

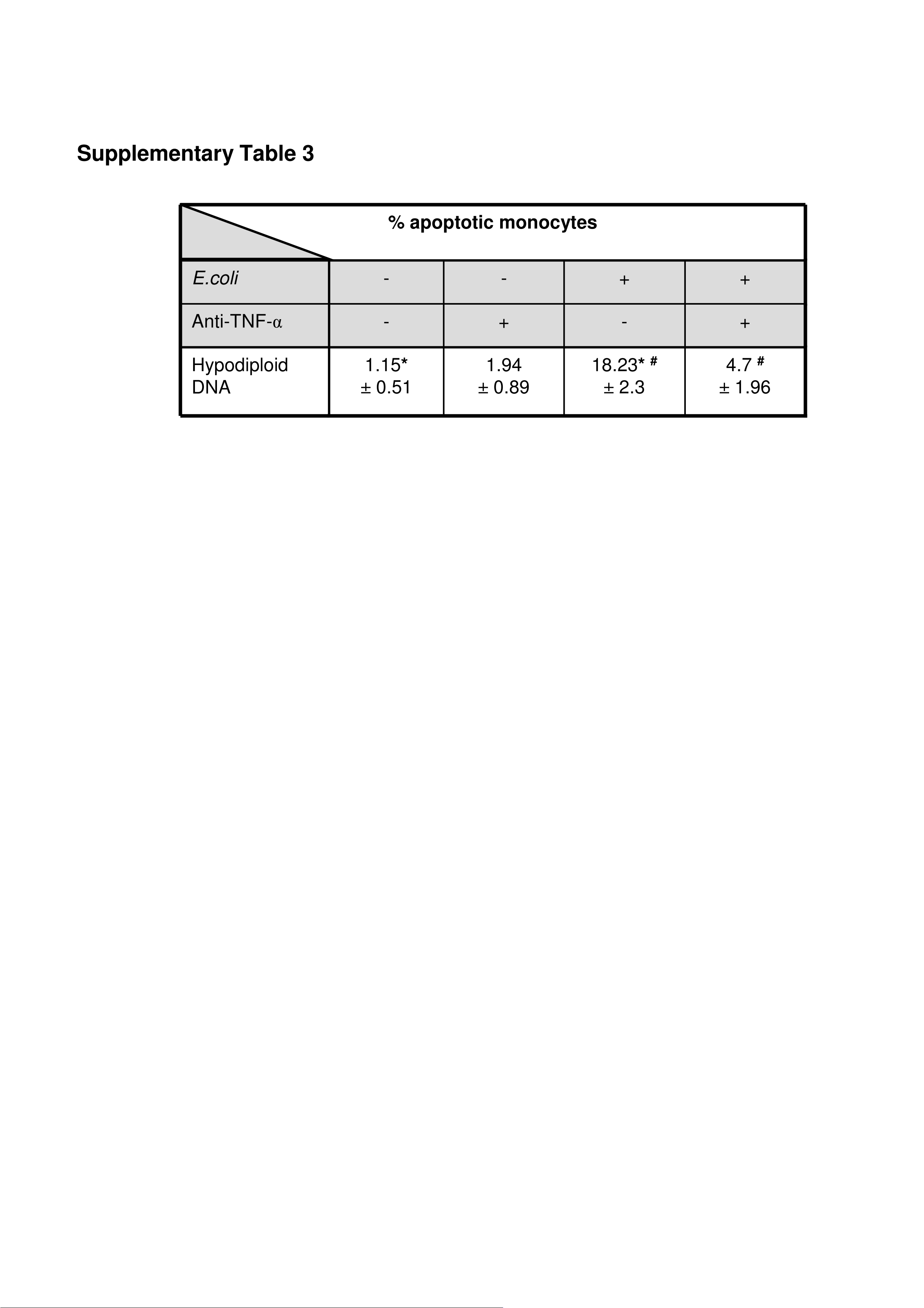

Supplement: Table S3 — aTNF-α mAb impairs monocyte apoptosis. Mononuclear cells were infected with E. coli-GFP for 240 minutes. One group received anti-TNF-α mAb in parallel to bacterial infection. Apoptosis detection (hypodiploid DNA-content) for all CD14+ monocytes and for the subsets of GFP+/CD14+ and GFP−/CD14+ (n = 5; *p<0.05 non-infected vs. infected, # p<0.05 infected w/o anti-TNF-α vs. infected w anti-TNF-α antibodies). (TIF) [file pone.0053589.s008.tif]
